# Impact of ibrutinib on inflammation in a mouse model of Graves’ orbitopathy

**DOI:** 10.3389/fendo.2024.1420024

**Published:** 2024-08-30

**Authors:** Charm Kim, Jin Hwan Park, Yeon Jeong Choi, Hyung Oh Jun, Jin Kwon Chung, Tae Kwann Park, Jin Sook Yoon, Jae Wook Yang, Sun Young Jang

**Affiliations:** ^1^ Department of Ophthalmology, Soonchunhyang University Cheonan Hospital, Soonchunhyang University College of Medicine, Cheonan, Republic of Korea; ^2^ Department of Ophthalmology, Soonchunhyang University Bucheon Hospital, Soonchunhyang University College of Medicine, Bucheon, Republic of Korea; ^3^ Department of Ophthalmology, Asan Hospital, The Institute of Vision Research, Eulji University College of Medicine, Seoul, Republic of Korea; ^4^ Department of Ophthalmology, Soonchunhyang University Seoul Hospital, Soonchunhyang University College of Medicine, Seoul, Republic of Korea; ^5^ Department of Ophthalmology, Severance Hospital, The Institute of Vision Research, Yonsei University College of Medicine, Seoul, Republic of Korea; ^6^ Department of Ophthalmology, Pusan Paik Hospital, Inje University College of Medicine, Busan, Republic of Korea

**Keywords:** Bruton’s tyrosine kinase, interleukin-2 inducible T-cell kinase, Graves’ orbitopathy, ibrutinib, inflammation, mouse

## Abstract

**Introduction:**

Bruton’s tyrosine kinase (BTK) and interleukin (IL)-2 Inducible T-cell Kinase (ITK) inhibitors have anti-inflammatory properties. We investigated the therapeutic effect of ibrutinib, an orally bioavailable BTK/ITK inhibitor, in a mouse model of Graves’ orbitopathy (GO).

**Methods:**

Genetic immunization was performed through intramuscular administration of the recombinant plasmid, pCMV6-hTSHR cDNA, to 8-week-old female BALB/c mice. Serum levels of T3, T4, and thyroid-stimulating hormone receptor (TSHR) antibodies (TRAbs) were quantified using enzyme-linked immunosorbent assay. Histopathological changes in orbital tissues were examined using immunohistochemistry (IHC) staining for TSHR and various inflammatory markers. Following successful genetic immunization, ibrutinib was orally administered daily for 2 weeks in the GO model mice. After treatment, the mRNA and protein expression levels of BTK, ITK, IL-1β, and IL-6 in orbital tissues were evaluated using real-time PCR and Western blotting.

**Results:**

In total, 20 mice were sacrificed to confirm successful genetic immunization. The GO mouse group exhibited significantly increased serum T3, T4, and TRAb levels. IHC revealed increased expression of TSHR, IL-1β, IL-6, transforming growth factor-β1, interferon-γ, CD40, CD4, BTK, and ITK in the GO mouse model. The orbital inflammation was significantly attenuated in ibrutinib-treated mice. The mRNA and protein expression levels of BTK, ITK, IL-1β, and IL-6 in orbital tissue were lower in ibrutinib-treated GO mouse group compared to the phosphate-buffered saline-treated GO mouse group.

**Conclusion:**

The GO mouse model demonstrated enhanced BTK and ITK expression. Ibrutinib, a BTK/ITK inhibitor, suppressed the inflammatory cytokine production. These findings highlight the potential involvement of BTK/ITK in the inflammatory pathogenesis of GO, suggesting its role as a novel therapeutic target.

## 1 Introduction

Graves’ orbitopathy (GO) is an autoimmune inflammatory disorder and significant extrathyroidal manifestation of Graves’ disease (GD) (1). Its etiology involves the presence of thyroid-stimulating hormone receptors (TSHRs) on orbital fibroblasts, which plays a key role in autoimmunity in GO (2, 3). TSHR is expressed by orbital fibroblasts and binds to anti-thyrotropin receptor antibodies (TRAbs), leading to inflammation, hyaluronan production, adipogenesis, and myofibrosis. In turn, this leads to inflammation, enlargement, and fibrosis of orbital connective tissues, including extraocular muscles (EOM) (2, 4, 5). However, the diverse symptoms in GO cannot be solely explained by this mechanism. The clinical spectrum of GO encompasses diverse manifestations, including eyelid swelling, retraction, dull pain, chemosis, conjunctival injection, proptosis, strabismus, diplopia, and compressive optic neuropathy (6, 7). The varied clinical presentations and severity of GO pose challenges in identifying its manifestations in individuals, leading to a lack of standardized treatment options.

Animal models of GO are crucial for understanding the complex pathophysiological processes of this condition and for developing new therapeutic approaches. While animal models are crucial tools for comprehending complex diseases, an optimal *in vivo* model for GO is currently lacking due to low reproducibility. Although efforts have been made to establish suitable animal models, the majority of basic experiments on GO have been conducted using *in vitro* models, culturing orbital fibroblasts collected directly from patients. Even though GO studies are still mainly being conducted using *in vitro* models of orbital fibroblasts, extensive efforts have been made to establish an animal model (8, 9). Two efficient techniques are available for establishing *in vivo* models of GD: adenovirus genetic immunization and plasmid genetic immunization. Moshkelgosha et al. (10) are credited with establishing the first true animal model of GO. More recently, Bao et al. (11) proposed a novel mouse model based on Cre-loxP system, capable of generating the hTSHR A-subunit following a single injection. Previous methods required repeated immunizations, so achieving successful immunization with a single injection represents a significant improvement in efficacy (11).

Bruton’s tyrosine kinase (BTK), a member of the TEC tyrosine kinase family, plays a major role in B-cell development, activation, differentiation, proliferation, and survival through the B-cell antigen receptor signaling pathway. Additionally, BTK is involved in Toll-like receptor and chemokine receptor signaling, regulating B cell adhesion and migration (12–14). Several therapeutic studies and clinical trials are being conducted to target BTK for the treatment of various autoimmune and inflammatory immune-mediated diseases, such as rheumatoid arthritis (RA), multiple sclerosis, systemic lupus erythematosus (SLE), Sjögren’s disease, and graft-versus-host disease. BTK inhibitors have anti-inflammatory properties. Initially approved as a BTK inhibitor, ibrutinib is orally bioavailable and is used to treat various B-cell malignancies and chronic graft-versus-host disease (15–19). However, ibrutinib can inhibit both BTK and IL-2 Inducible T-cell Kinase (ITK) impairing B-cell and T-cell function. The homology between BTK and ITK makes ITK an off-target kinase inhibited by ibrutinib (20). ITK is an enzyme responsible for the phosphorylation and activation of downstream effectors in T-cell (TCR) signaling pathway (21).

Our study group recently investigated the therapeutic effect of ibrutinib in the pathogenesis of GO using an *in vitro* model. By characterizing the role of BTK in an *in vitro* model of GO, we found that BTK expression was higher in GO tissues than in normal tissues. Moreover, ibrutinib suppressed the interleukin (IL)-1β- and insulin-like growth factor (IGF)-1-induced proinflammatory cytokines, including IL-6, IL-8, and cyclooxygenase-2 (COX-2), in both GO and normal orbital fibroblasts (22). Also, we investigated the effect of selective ITK inhibitor on inflammation in an *in vitro* model of GO (23). As an extension of our previous research, this study investigated the therapeutic efficacy of ibrutinib in a GO mouse model.

## 2 Materials and methods

### 2.1 Human blood sample preparation

We enrolled 15 patients with GO and 15 controls without GO who presented to Soonchunhyang University Hospital between January 2020 and February 2022. Whole blood was collected in PAXgene Blood RNA tubes (Becton Dickinson and Company, Franklin Lakes, NJ, USA) from both patient groups and the control groups and subsequently aliquoted and stored at −80°C until analysis. The study participants provided written informed consent. The study protocol was approved by the Institutional Review Board of Soonchunhyang Bucheon Hospital, Soonchunhyang University College of Medicine (2020-04-006). The study was conducted in accordance with the Declaration of Helsinki. **Table 1** presents the detailed clinical characteristics of the participants.

**Table 1:** Demographic characteristics of the participants.

	Graves’ orbitopathy (*n* = 15)	Controls (*n* = 15)	*p*-value
Age (years)	43.87 ± 10.54	43.4 ± 12.80	0.904
Sex (female)	10 (66.7)	11 (73.3)	0.690
Smoker	5 (33.3)	3 (20.0)	0.409
Diabetes	0 (0)	0 (0)	NA
Hypertension	1 (6.7)	2 (13.3)	0.543
Duration (years)	3.48 ± 2.24	NA	NA
Clinical activity score	1.6 ± 1.18	NA	NA

The data is presented as mean ± standard deviation or n (%).

NA, not available.

### 2.2 Animal preparation

**Figure 1:**
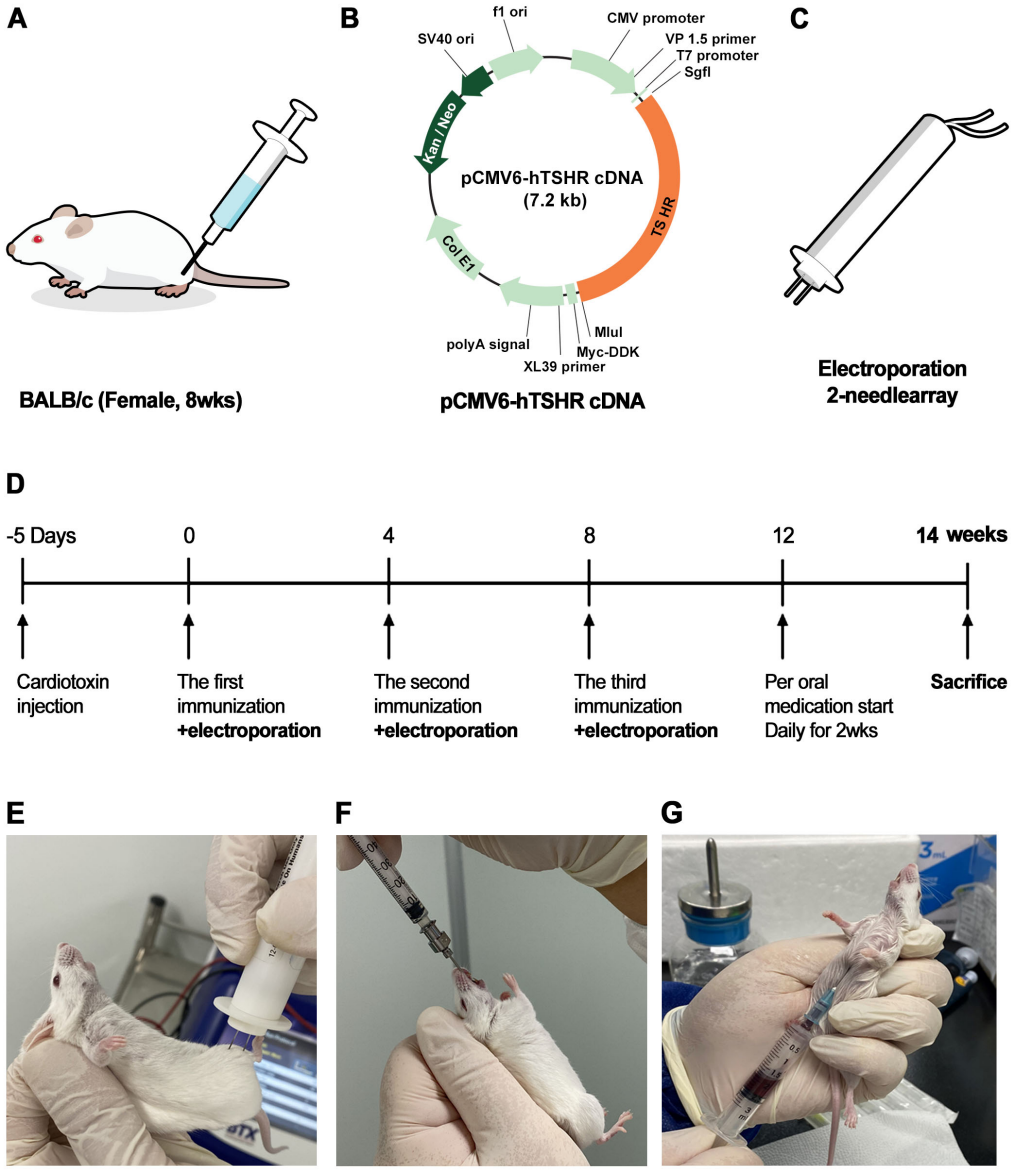
Flowchart for the generation of pCMV6-hTSHR complementary deoxyribonucleic acid (cDNA)-induced Graves’ orbitopathy (GO) model mice **(A–E)**. Ibrutinib administration and sacrifice of genetically immunized mice **(F, G)**. **(A)** To induce an autoimmune condition and perform genetic immunization, cardiotoxin (CTX) was administered to 8-week-old female BALB/c mice. **(B)** The recombinant plasmid pCMV6-hTSHR cDNA was injected into the biceps femoris muscle. **(C, E)** Electroporation with an ECM 830 system and 100 mm electrode needle was performed at 200 V/cm immediately following CTX administration. **(D)** The vaccination process was repeated 3 times at 4-week intervals.

In total, 30 BALB/c female mice aged 6 weeks were obtained from Raonbio (Yong-in, Korea). Animals were housed under conventional conditions in cages with filter lids, with *ad libitum* access to food. The animal experiments were conducted in accordance with the Association for Research in Vision and Ophthalmology guidelines for the Use of Animals in Ophthalmic and Vision Research. The study protocol was approved by the Institutional Animal Care and Use Committee at Soonchunhyang University Bucheon Hospital. Twenty mice were sacrificed to validate successful genetic immunization. Of these mice, 10 received immunization with the hTSHR plasmid, whereas the remaining 10 mice served as the control group (pCMV6-entry). After confirming successful genetic immunization, we immunized 10 additional BALB/c mice with pCMV6-hTSHR cDNA. Of these mice, five were treated with ibrutinib (10 mg/kg/day (21),), whereas the remaining five were treated with phosphate-buffered saline (PBS) administered intragastrically every day for 2 weeks (**Figure 1F**). Ibrutinib used were purchased from: Selleckchem and dissolved in dimethyl sulfoxide (DMSO) according to manufacturer’s instruction (24). Briefly, ibrutinib was completely dissolved in 1mL of DMSO to prepare a 10mg/mL solution, which was then diluted with water to create a 2 mg/mL solution. Given that the mice weigh approximately 20-25 g each, a dose of about 200 μg/100 μL per mouse was administered.

### 2.3 Plasmid construction

The pCMV6-hTSHR cDNA was obtained from OriGene Technologies, Inc. (Rockville, MD, USA). **Figure 1B** provides schematic diagrams of plasmid pCMV6-hTSHR. The plasmids were cultured in *Escherichia coli* XL-1 Blue cells in Luria broth medium and purified using the Plasmid Maxi Kit (Qiagen, Hilden, Germany). The concentration of the purified plasmid was evaluated using a Nanodrop spectrophotometer, resuspended at 1 mg/mL in sterile water, and stored at −80°C.

### 2.4 Genetic immunization by DNA vaccination

Genetic immunization was performed by intramuscular administration of the recombinant plasmid, pCMV6-hTSHR cDNA, three times in female 8-week-old BALB/c mice (**Figures 1A, B**). Then, *in vivo* electroporation was performed using the ECM 830 square wave electroporator (BTX Harvard Apparatus, Holliston, MA, USA) (**Figure 1C**). To induce an autoimmune condition, the biceps femoris muscle was injected with 10 μM cardiotoxin (CTX; Naja nigricollis, 10 µM; Calbiochem, La Jolla, CA, USA). Five days after CTX inoculation, 100 µg/100 µL of plasmid DNA was injected into the biceps femoris muscle. The vaccination was repeated three times at 4-week intervals (**Figure 1D**). Each vaccination was immediately followed by electroporation using an ECM 830 system with 100-mm electrode needles at 200 V/cm (**Figure 1E**). The current was applied in 10 20-ms square wave pulses at 1 Hz, causing marked muscle twitching.

Twenty mice were divided into control (pCMV6-entry) (*n* = 10) and TSHR (pCMV6-hTSHR) DNA immunization (*n* = 10) groups. After confirming successful genetic immunization, we conducted immunization with pCMV6-hTSHR cDNA on 10 additional BALB/c mice.

### 2.5 Mouse blood and orbital tissue sample collection

To confirm the successful genetic immunization and investigate the effect of the BTK/ITK dual inhibitor ibrutinib (10 mg/kg/day) on orbital inflammation, the mice were humanely sacrificed, and blood and orbital fat tissue were collected. Blood was collected intracardiacally using a 1-cc syringe (**Figure 1G**). Enucleation of both eyes was performed, and orbital whole tissue from the retrobulbar space was obtained after sacrifice.

### 2.6 Serological analysis of mouse blood

The mouse serum levels of T3, T4, and TRAb were assessed by ELISA. Mouse serum T3 and T4 levels were evaluated using a mouse T3 ELISA kit (#OKA00141; Aviva Systems Biology, San Diego, CA, USA) and a mouse T4 ELISA kit (#OKA00140; Aviva Systems Biology). Mouse serum TSHR-ab level was measured using a mouse TSHR-ab ELISA kit (#MBS2505459; MyBioSource Inc., San Diego, CA, USA) according to the manufacturer’s instructions.

### 2.7 Histological analysis and immunohistochemistry

For histological evaluation, orbital tissues were fixed in 4% paraformaldehyde, embedded in paraffin, and cut into 4-μm sections. The sections were stained with hematoxylin and eosin (H&E) for evaluation of pathological changes. Additionally, IHC staining was performed using the TSHR and several inflammatory markers. For IHC analysis of the TSHR, confocal microscopy images were obtained to visualize the TSHR (green) and live cell nuclei (Hoechst; blue) in mouse orbital tissues. Deparaffinization was performed by incubating the sections twice in xylene, followed by incubation in 100% ethanol, for 5 min each. Then, the sections were incubated with PBS containing 0.1% Triton X-100 for 10 min to enhance antibody penetration. After blocking with 5% normal goat serum in PBS for 30 min at room temperature, the samples were incubated with primary antibodies, including anti-TSHR antibody (1:100, #ab27974; Abcam, Cambridge, UK) in 1% bovine serum albumin and PBS containing 0.1% Triton X-100 for 1 h at room temperature or overnight at 4°C. The sections were washed with PBS three times, with each wash lasting 5 min. Then, the tissue was incubated with the secondary antibody in 1% bovine serum albumin for 1 h at room temperature in the dark. Subsequently, the secondary antibody solution was decanted, and the sections were washed with PBS three times for 5 min each. Nuclear counterstaining was performed using Hoechst 33258 (2.5 μg/mL, H1399; Thermo Fisher Scientific, Waltham, MA, USA) in the dark at room temperature for 15 min. Images were captured using confocal fluorescence microscopy (LSM710; Carl Zeiss, Oberkochen, Germany). Alexa Fluor-488 antimouse (1:1000, A-11001; Thermo Fisher Scientific) and Alexa Fluor-594 antirabbit (1:1000, A-11012; Thermo Fisher Scientific) antibodies were used as the secondary antibodies.

For IHC analysis of several inflammatory markers, paraffin-embedded sections underwent deparaffinization and dehydration. After washing with PBS, endogenous peroxidase activity was quenched with 3% H_2_O_2_ in methanol, followed by blocking of non-specific binding sites using 5% normal goat serum in PBS. Then, the sections were incubated for 1 h with BTK (#ab208937; AbTGFcam), ITK (#CST2380; Cell Signaling Technology, Danvers, MA, USA), CD4 (#ab183685; Abcam), CD40 (#ab13545; Abcam), IL-1β (#ab9722; Abcam), transforming growth factor-β1 (TGF-β1; #ab215715; Abcam), IL-6 (#GTX110527; GeneTex, Irvine, CA, USA), and interferon-γ (IFN-γ; #MM700; Invitrogen, Carlsbad, CA, USA). After several washes, the samples were processed for color development using the DAB Substrate Kit (#SK-4100; Vector Laboratories, Burlingame, CA, USA), according to the manufacturer’s protocols. The slides were counterstained with hematoxylin for 10 s, followed by dehydration in increasing concentrations of ethanol and clarification with xylene. The slides were covered with Limonene mounting medium and a cover glass.

To quantification of IHC results, the IHC toolbox from ImageJ soft software (National Institutes of Health, Bethesda, MD, USA [in the public domain]) was used.

### 2.8 Quantitative real-time polymerase chain reaction

Levels of BTK, ITK, phospholipase C-γ (PLC-γ)1 and (PLC-γ)2 in human blood were assessed by qRT-PCR. RNA in human blood was extracted from PAXgene tubes using the Paxgene Blood RNA kit (Becton Dickinson and Company). Total RNA was isolated using TRIzol (#AM9738; Ambion, Austin, TX, USA) and reverse-transcribed into complementary DNA using a cDNA reverse transcription kit (High Capacity cDNA Reverse Transcription Kit; #4368814; Applied Biosystems, Waltham, MA, USA). The resulting cDNA was amplified using a thermocycler (ABI, Foster City, CA, USA) with a PowerUp SYBR Green Master Mix (#A25918; ABI) and universal PCR master mix (TaqMan No AmpErase UNG; #4324018; ABI). PCR was performed according to the manufacturer’s recommendations. The mRNA expression was standardized relative to the glyceraldehyde-3-phosphate dehydrogenase (GAPDH) expression. PCR amplification was performed using specific primer pairs presented in (**Table 2**).

**Table 2:** Primer sequences used in this study.

Gene name	Type	Sequences (5’-3’)
BTK	Forward Reverse forward	GGCTCCAAGTTTCCAGTCCG AACCCCAAAAGCCCAGATGTC
ITK	Forward Reverse forward	CGCTACTACGTGGCTGAGAAGT CAGGAGCAAACTGGATAGCGGA
IL-1β	Forward Reverse forward	GCACTACAGGCTCCGAGATGAA GTCGTTGCTTGGTTCTCCTTGT
IL-6	Forward Reverse forward	TCCTTCCTACCCCAATTTCCA GTCTTGGTCCTTAGCCACTCC
TGF-β1	Forward Reverse forward	CTCCCGTGGCTTCTAGTGC GCCTTAGTTTGGACAGGATCTG
IFN-γ	Forward Reverse forward	GCTACACACTGCATCTTGGC TTTCAATGACTGTGCCGTGG
CD40	Forward Reverse forward	ACCAGCAAGGATTGCGAGGCAT GGATGACAGACGGTATCAGTGG
CD4	Forward Reverse forward	TCCCACTCACCCTCAAGATA ATCACCACCAGGTTCACTTC
PLC-γ1	Forward Reverse forward	AGTTTGTGGTGGACAATGGACT ATACACCACGAAGCGCAGAA
PLC-γ2	Forward Reverse forward	GCGTCTACCCAAAGGGACAA GCCGTCTGGAAATTGAGTGC
GAPDH	Forward Reverse forward	TGCAGTGGCAAAGTGGAGATT TTGAATTTGCCGTGAGTGGA

BTK, Bruton’s tyrosine kinase; ITK, interleukin-2-inducible T-cell kinase; IL-1β, Interleukin-1beta; IL-6, Interleukin-6; TGF-β1, Transforming Growth Factor-beta1; IFN-γ, Interferon gamma; CD40, cluster of differentiation40; CD4, cluster of differentiation4 PLC-γ1, phospholipase C-gamma 1; PLC-γ2, phospholipase C-gamma 2; GAPDH, glyceraldehyde-3-phosphate dehydrogenase.

### 2.9 Western blot

Orbital fat tissues from euthanized mice were washed with ice-cold PBS, homogenized in the lysis buffer (RIPA II lysis buffer; GenDEPOT, #R4200-010), and incubated on ice for 1 h. The lysates were centrifuged at 12,000 × g for 10 min, and the tissue homogenate fractions were stored at −70°C until use. Protein concentrations were determined using the BCA protein assay. Equal quantities of protein (20 μg) were boiled in the sample buffer and dissolved using 10% (wt/vol) sodium dodecyl sulfate–polyacrylamide gel electrophoresis. Proteins were transferred onto polyvinylidene difluoride membranes (Immobilon; Millipore, Bedford, MA, USA). The samples were incubated overnight with primary antibodies (BTK; #ab208937, Abcam, ITK; #CST2380, Cell Signaling Technology, PLCG1; #CST2822, PLCG2; #CST3972, IL-1β; #ab9722, IL-6; #GTX110527, GeneTex, IFN-γ; #MM700, Invitrogen, TGF-β1; #ab215715, CD40; #ab13545, and CD4; #ab183685) in Tris-buffered saline containing Tween 20 and 5% skim milk or 5% bovine serum albumin. Subsequently, the specimens were washed three times with TBS and 0.1% Tween 20 buffer. Immunoreactive bands were detected using horseradish peroxidase-conjugated secondary antibody and developed using an enhanced chemiluminescence kit (#W3652; GenDepot, Barker, TX, USA). The immunoreactive bands were quantified using densitometry, and their levels were normalized to the β-actin level in the same sample.

### 2.10 Data analyses

The number of technical replicates for IHC and WB, as well as the number of mice used for all experiments, is specified in the respective results sections and figure legends. The results are presented as means ± standard deviations (SDs) of normalized measurements. Differences between groups were assessed by independent *t-*tests and one-way analysis of variance with *post-hoc* analysis using SPSS software (version 26.0; IBM Corp., Armonk, NY, USA). All *p*-values of less than 0.05 were considered indicative of statistical significance.

## 3 Results

### 3.1 Demographic characteristics

Blood samples were obtained from 15 patients with GO and 15 age- and sex-matched controls who did not have a history of GO. **Table 1** presents the demographic characteristics of the participants. The mean age of GO patients was 43.87 ± 10.54 years, and 10 (66.7%) were female. The mean age of controls was 43.4 ± 12.8 years, and 11 (73.3%) were females. There were five and three smokers in the GO and non-GO groups, respectively.

### 3.2 Increased serum levels of BTK and ITK in GO patients

**Figure 2:**
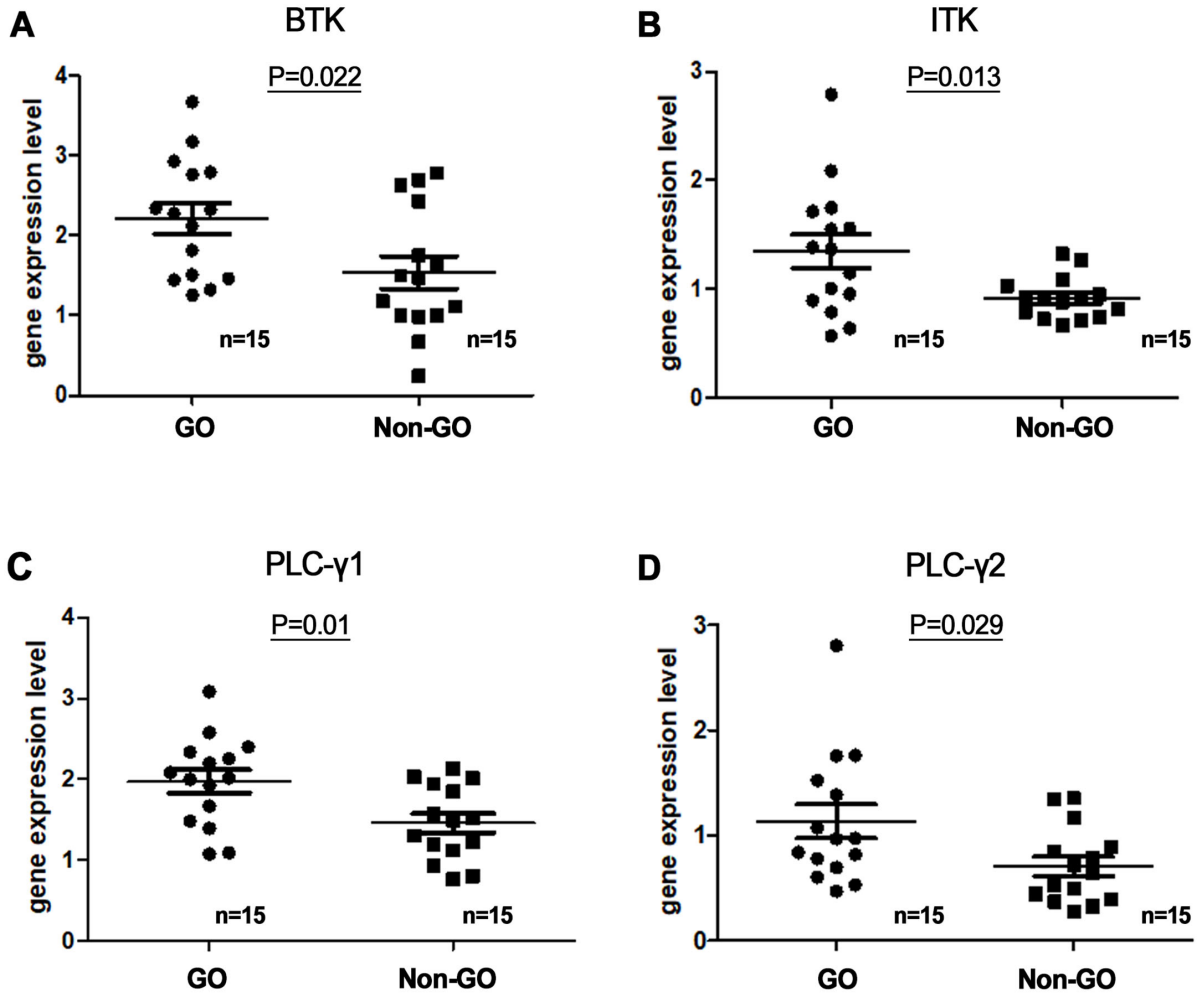
Serum messenger ribonucleic acid (mRNA) expression levels of **(A)** Bruton’s tyrosine kinase (BTK), **(B)** interleukin-2-inducible T-cell kinase (ITK), and **(C, D)** phospholipase C-γ (PLC-γ) 1 and 2 in Graves’ orbitopathy (GO) patients (n=15) and non-GO individuals (n=15). BTK, ITK, PLC-γ1, and PLC-γ2 transcription levels were assessed using reverse transcription-polymerase chain reaction. mRNA expression levels of BTK, ITK, PLC-γ1, and PLC-γ2 were higher in GO patients (2.21 ± 0.19, 1.35 ± 0.15, 1.97 ± 0.14, and 1.13 ± 0.16, respectively) than non-GO individuals (1.54 ± 0.20, 0.92 ± 0.05, 1.46 ± 0.12, and 0.76 ± 0.09, respectively). (p=0.022, 0.013, 0.01, and 0.029, respectively).

To determine whether the BTK pathway is involved in the pathogenesis of GO, we compared the serum levels of BTK, ITK, PLCγ1, and PLCγ2 between the GO (*n* = 15) and non-GO (*n* = 15) groups using reverse transcription-PCR (RT-PCR). *In vivo* experiments were performed by extracting mRNA from the blood of GO patients and controls. RT-PCR was used to compare the BTK, ITK, PLC-γ1, and PLC-γ2 transcription levels between GO and normal tissues (**Figure 2**), revealing significantly higher baseline serum expression levels of BTK, ITK, PLC-γ1, and PLC-γ2 in the GO group (*n* = 15) than in the control group (*n* = 15). mRNA expression levels of BTK, ITK, PLC-γ1, and PLC-γ2 were higher in GO patients (2.21 ± 0.19, 1.35 ± 0.15, 1.97 ± 0.14, and 1.13 ± 0.16, respectively) than non-GO individuals (1.54 ± 0.20, 0.92 ± 0.05, 1.46 ± 0.12, and 0.76 ± 0.09, respectively). (p=0.022, 0.013, 0.01, and 0.029, respectively).

### 3.3 Successful induction of GO mouse model

**Figure 3:**
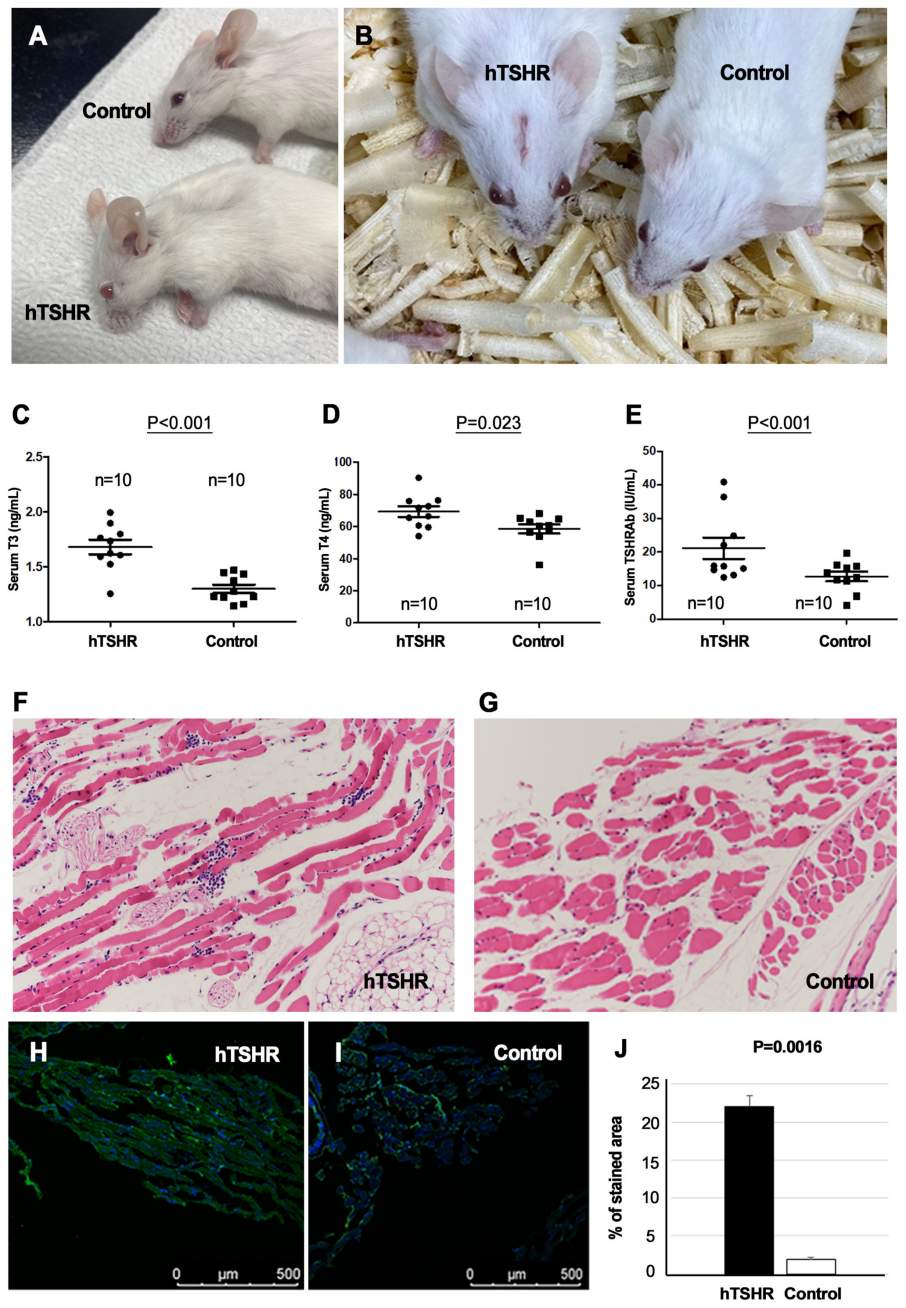
Phenotype, serum levels, and pathological analysis of Graves’ orbitopathy (GO) mice after successful immunization. **(A, B)** Mice immunized with the human thyrotropin hormone receptor (TSHR) plasmid exhibited significant proptosis. **(C–E)** Serum levels of T3, T4, and thyrotropin hormone receptor TSHR antibodies (TRAbs) were significantly higher in the GO mouse group (n=10) compared to the control group (n=10). **(F, G)** Hematoxylin–eosin (H&E) staining revealed retrobulbar inflammation of extraocular muscles and adipogenesis in human TSHR plasmid-immunized mice. Photos of H&E staining show 1 mice/group and are representative of 1 independent experiments with samples from 3 mice per group. **(H–J)** Immunohistochemistry (IHC) demonstrated increased hTSHR expression in the GO mouse model (22.1 ± 1.36% of area) compared to non-GO mouse model (1.9 ± 0.28% of area) (p=0.0016). **(H, I)** Photos of IHC show 1 mice/group and are representative of 1 independent experiments with samples from 3 mice per group. **(J)** Bar graphs use data from all 3 mice per group.

In total, 20 female BALB/c mice were sacrificed to validate the successful genetic immunization (**Figure 1**). Of these mice, 10 were immunized with the hTSHR plasmid, whereas the remaining 10 were immunized with the control plasmid. Half of the hTSHR plasmid-immunized mice had significant proptosis (**Figures 3A, B**). Serum T3, T4, and TRAb levels were significantly higher in the GO group (n=10) than in the control group (n=10) (**Figures 3C–E**). H&E staining revealed retrobulbar inflammation of extraocular muscles and adipogenesis in tissues obtained from hTSHR plasmid-immunized mice (**Figures 3F, G**). IHC analysis demonstrated higher hTSHR expression in the GO mouse model (22.1 ± 1.36% of area) compared to the control group (1.9 ± 0.28% of area) (p=0.0016) (**Figures 3H–J**).

**Figure 4:**
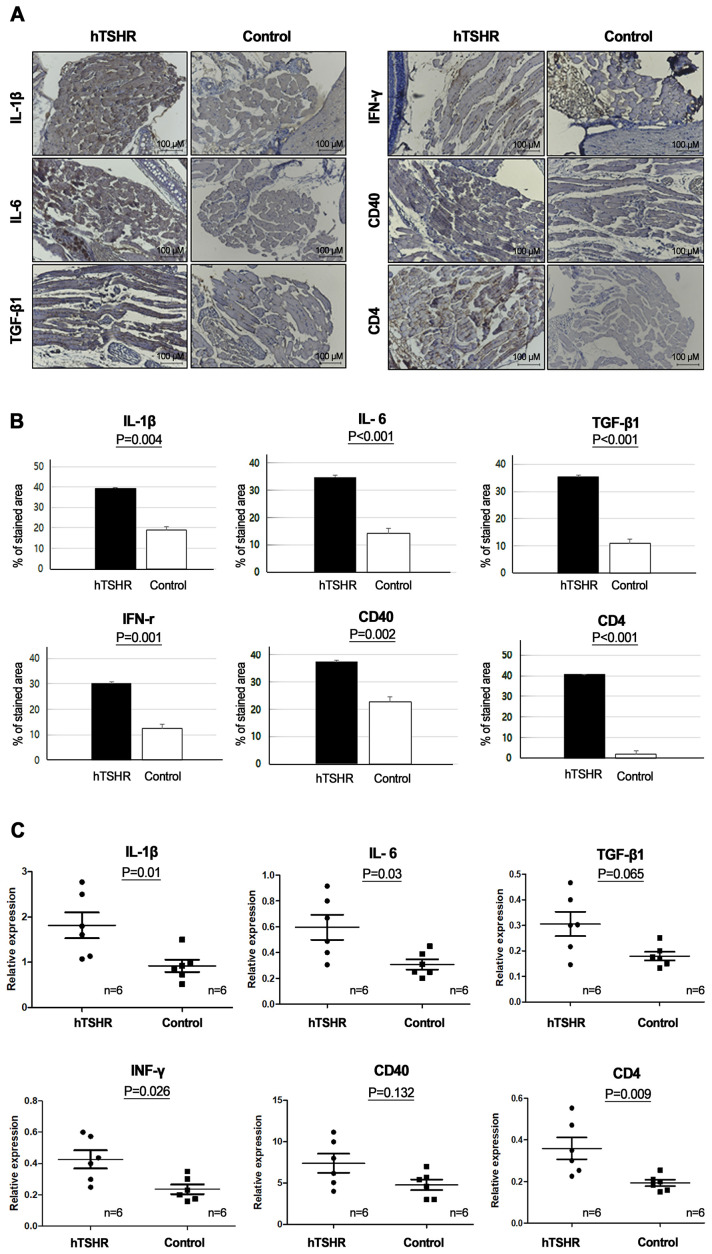
Inflammatory changes in the retrobulbar tissue of the Graves’ orbitopathy (GO) mouse model. **(A, B)** Immunohistochemistry (IHC) revealed higher expression levels of interleukin (IL)-1β (p=0.04), IL-6 (p<0.001), transforming growth factor-β1 (TGF-β1), (p<0.001), interferon-γ (IFN-γ) (p=0.001), cluster of differentiation (CD) 40 (p=0.002), and CD4 (p<0.001) in the GO mouse model. **(A)** Photos of IHC show 1 mice/group and are representative of 1 independent experiments with samples from 3 mice per group. **(B)** Bar graphs use data from all 3 mice per group. **(C)** Reverse transcription-polymerase chain reaction (RT-PCR) demonstrated higher messenger ribonucleic acid expression levels of IL-1β (p=0.01), IL-6 (p=0.03), TGF-β1(p=0.065), IFN-γ (p=0.026), CD40 (p=0.132), and CD4 (p=0.009), in the GO mouse model (n=6) compared to the controls (n=6). Statistically significant differences were observed in all inflammatory cytokines, except TGF-β and CD40.

Furthermore, the inflammatory changes in the retrobulbar tissue of the GO mouse model were confirmed with IHC staining and qRT-PCR of several inflammatory markers, such as IL-1β, IL-6, TGF-β1, IFN-γ, CD40, and CD4. IHC analysis showed statistically significant higher expression levels of IL-1β (p=0.04), IL-6 (p<0.001), TGF-β1, (p<0.001), IFNγ (p=0.001), CD40 (p=0.002), and CD4 (p<0.001) in the GO mouse model (39.10 ± 0.64, 34.92 ± 0.94, 35.37 ± 0.95, 30.15 ± 1.26, 37.40 ± 0.59, and 40.50 ± 0.13 [% of area], respectively) compared to the controls (18.85 ± 1.65, 14.35 ± 0.53, 10.88 ± 0.72, 12.33 ± 0.18, 22.83 ± 0.97, and 1.98 ± 0.11 [% of area], respectively) (**Figures 4A, B**).

RT-PCR also revealed higher expression levels of IL-1β (p=0.01), IL-6 (p=0.03), TGF-β1 (p=0.065), IFNγ (p=0.026), CD40(p=0.132), and CD4 (p=0.009) in the GO mouse model (1.82 ± 0.29, 0.60 ± 0.10, 0.31 ± 0.05, 0.43 ± 0.06, 7.40 ± 1.15, and 0.36 ± 0.05, respectively) (n=6) compared to the controls (0.92 ± 0.13, 0.31 ± 0.04, 0.18 ± 0.02, 0.24 ± 0.03, 4.79 ± 0.65, and 0.19 ± 0.02, respectively) (n=6) (**Figure 4C**).

### 3.4 BTK and ITK signaling protein and mRNA expression in GO and non-GO mouse models

**Figure 5:**
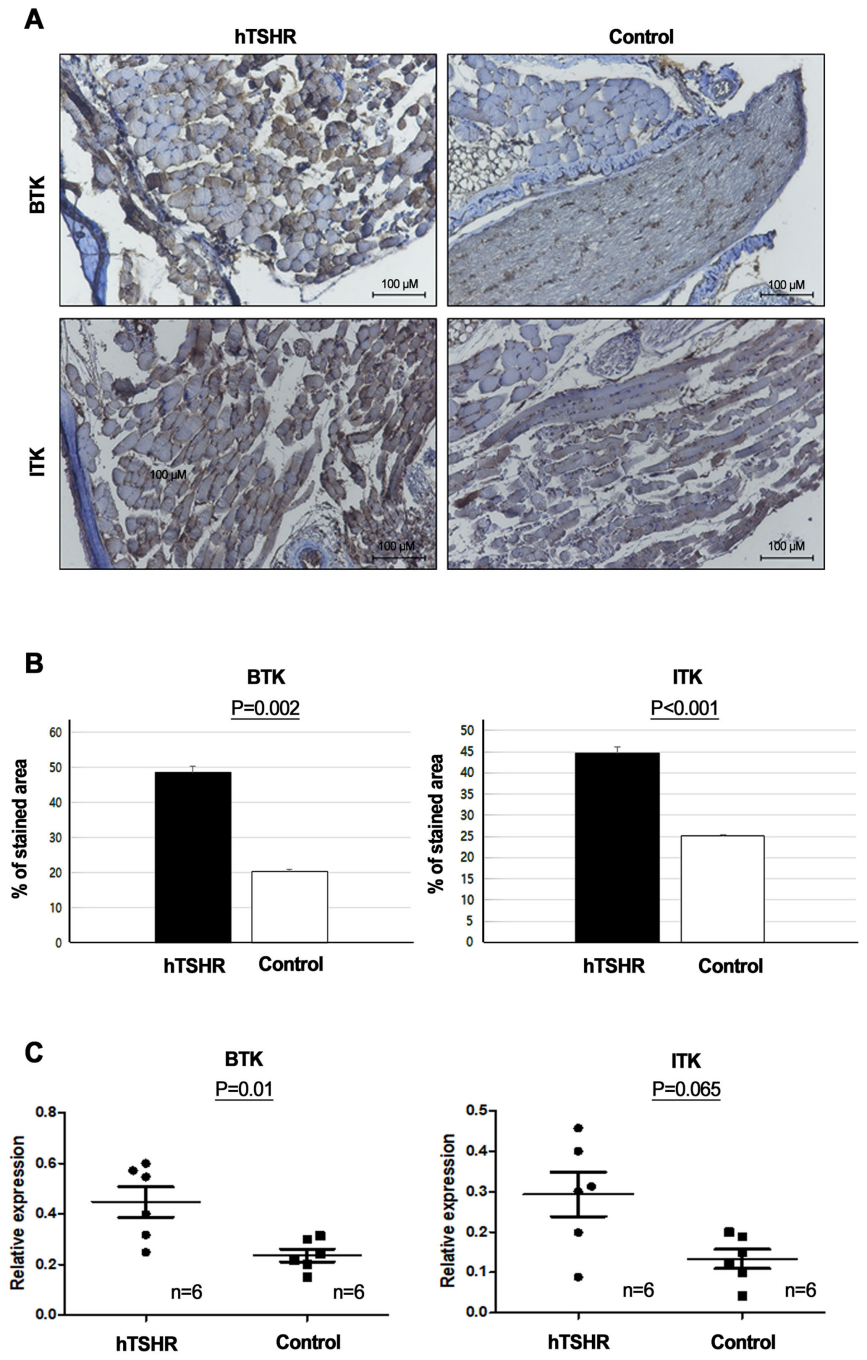
Comparison of Bruton’s tyrosine kinase (BTK) and interleukin-2-inducible T-cell kinase (ITK) signaling protein and mRNA expression levels between Graves’ orbitopathy (GO) and non-GO mouse models. **(A, B)** Immunohistochemistry **(IHC)** revealed elevated expression of BTK (p=0.002) and ITK (p<0.001) in the GO mouse model. **(A)** Photos of IHC show 1 mice/group and are representative of 1 independent experiments with samples from 3 mice per group. **(B)** Bar graphs use data from all 3 mice per group. **(C)** BTK (p=0.01) and ITK (p=0.065) expression levels were higher in human thyrotropin hormone receptor plasmid-immunized mice (n=6) compared to control plasmid-immunized mice (n=6).

To determine whether BTK and ITK expression were altered in the GO mouse model, we compared the expression levels of BTK and ITK in the orbital tissue between GO and non-GO mouse models using IHC staining and qRT-PCR. IHC revealed higher BTK (p=0.002) and ITK expression levels (p<0.001) in the GO mouse model (48.76 ± 1.54 and 44.60 ± 0.54 [% of area], respectively) compared to controls (20.47 ± 0.45 and 25.07 ± 0.13 [% of area], respectively) (**Figures 5A, B**).

Furthermore, RT-PCR demonstrated that the BTK (p=0.01) and ITK mRNA expression levels (p=0.065) were higher in the GO mouse model (0.45 ± 0.06 and 0.24 ± 0.03, respectively) (n=6) compared to non-GO mouse model (0.29 ± 0.05 and 0.13 ± 0.02, respectively) (n=6) (**Figure 5C**).

### 3.5 Effects of BTK/ITK inhibitor on BTK and ITK expression in the GO mouse model

**Figure 6:**
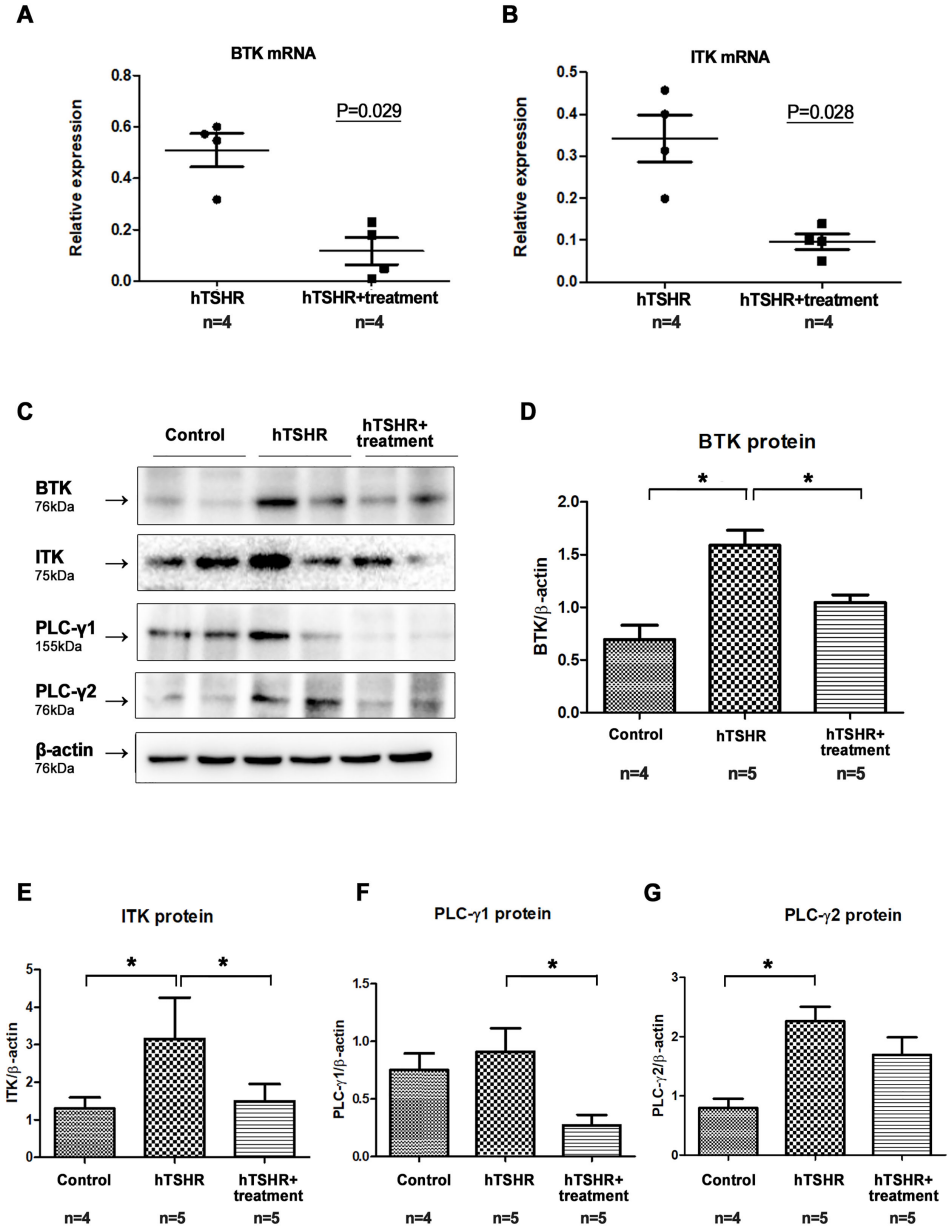
Effects of the Bruton’s tyrosine kinase (BTK) and interleukin-2-inducible T-cell kinase (ITK) inhibitor on BTK and ITK expression in the Graves’ orbitopathy (GO) mouse model. **(A, B)** Following treatment with the BTK/ITK inhibitor ibrutinib, the BTK and ITK transcription levels were observed to be reduced. RT-PCR demonstrated that the BTK (p=0.029) and ITK (p=0.028) mRNA expression levels were lower in the ibrutinib-treated GO mouse model (0.50 ± 0.06 and 0.34 ± 0.06, respectively) (n=4) compared to PBS-treated GO mouse model (0.12 ± 0.05 and 0.09 ± 0.02, respectively) (n=4) **(C–G)** Western blotting demonstrated elevated expression levels of BTK, ITK, phospholipase C-γ (PLC-γ)1, and PLC-γ2 in the GO mouse model. Notably, the increased expression of BTK, ITK, PLC-γ1, and PLC-γ2 in the GO mouse model was suppressed by ibrutinib. WB show 2 mice/group and are representative of 2 independent experiments with samples from 4 control mice and 5 GO mouse and 5 ibrutinib-treated GO mouse group. **(B)** Bar graphs use data from all mice per group. ^*^ *p*<0.05.

After confirming successful genetic immunization, we immunized 10 additional BALB/c mice with pCMV6-hTSHR cDNA. Of these mice, five were treated with ibrutinib, whereas the remaining five were treated with PBS for 2 weeks. We investigated the BTK and ITK mRNA expression levels in the orbital tissue of the GO mouse model after treatment with the ibrutinib. As a result, ibrutinib attenuated BTK and ITK transcription (**Figures 6A, B**). RT-PCR demonstrated that the BTK (p=0.029) and ITK (p=0.028) mRNA expression levels were lower in the ibrutinib-treated GO mouse model (0.50 ± 0.06 and 0.34 ± 0.06, respectively) (n=4) compared to PBS-treated GO mouse model (0.12 ± 0.05 and 0.09 ± 0.02, respectively) (n=4) (**Figures 6A, B**).

After confirming the suppressive effect of ibrutinib on BTK and ITK transcription, we evaluated its effect on the protein expression levels of BTK, ITK, PLC-γ1, and PLC-γ2. Western blotting revealed that the expression levels of BTK, ITK, PLC-γ1, and PLC-γ2 were higher in GO mouse model (n=5), compared to non-GO mouse model (n=4) (**Figures 6C-G**). However, in the ibrutinib-treated GO mouse model (n=5), the expression levels of BTK, ITK, PLC-γ1, and PLC-γ2 were lower compared to PBS-treated GO mouse model (n=5), indicating suppression by ibrutinib (**Figures 6C-G**, **Supplementary Figure 1**). Representative photos of two independent samples from each group (non-GO mice, PBS-treated GO mice, ibrutinib-treated GO mice) were provided (**Figure 6C**).

### 3.6 Effects of BTK/ITK inhibitor on inflammation in GO mouse model

**Figure 7:**
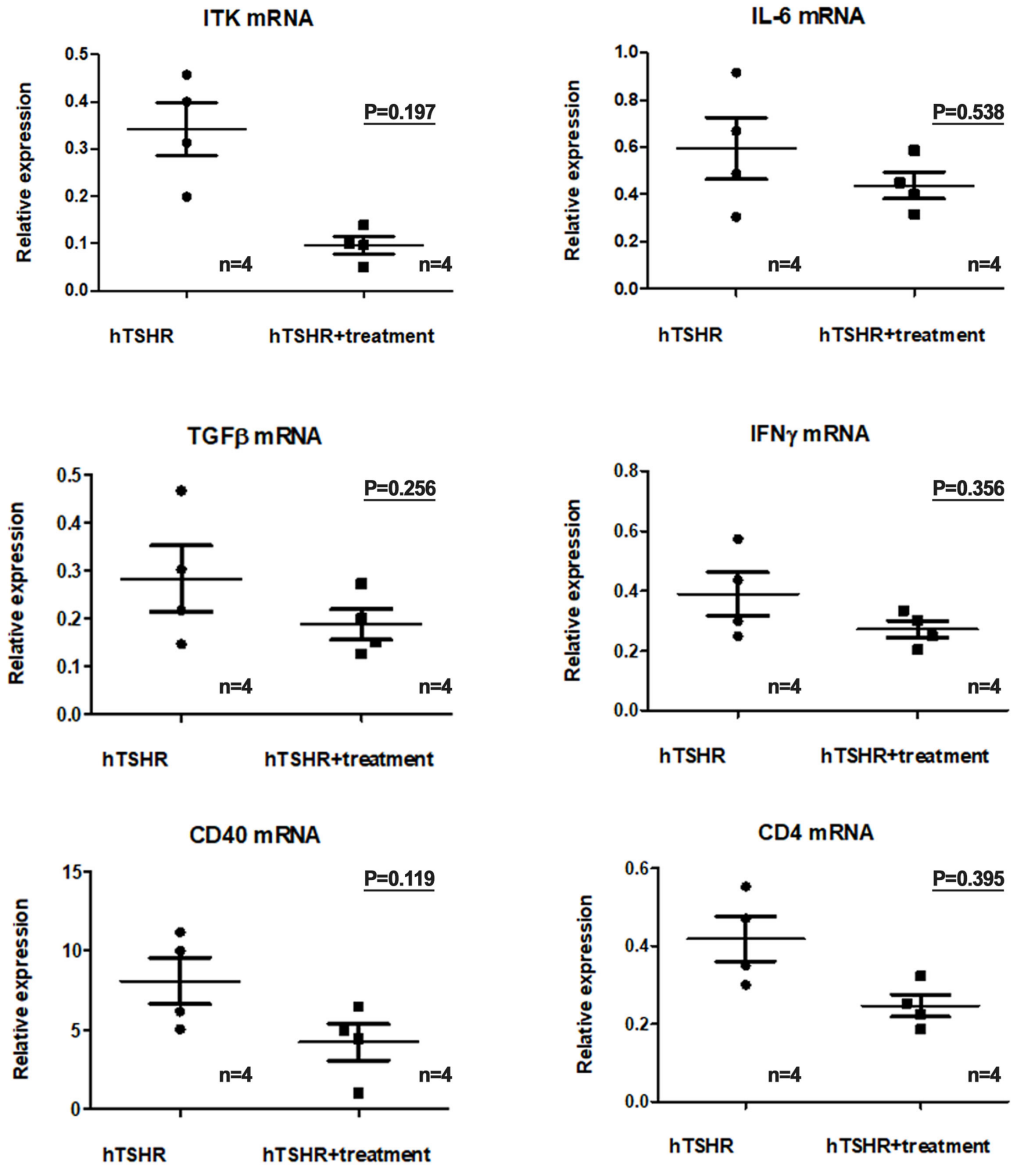
Effects of the Bruton’s tyrosine kinase (BTK) and interleukin-2-inducible T-cell kinase (ITK) inhibitor on the transcription of proinflammatory cytokines in the Graves’ orbitopathy (GO) mouse model. Reverse transcription-polymerase chain reaction revealed a decrease in the messenger ribonucleic acid expression levels of proinflammatory cytokines, including interleukin (IL)-1β (p=0.197), IL-6 (p=0.538), transforming growth factor-β1 (p=0.256), interferon-γ (p=0.356), cluster of differentiation (CD)40 (p=0.119), and CD4 (p=0.395), in the GO mouse model following treatment with the BTK/ITK inhibitor ibrutinib. However, these changes did not reach statistical significance. Each group in this experiment consisted of 4 mice.

We investigated changes in the mRNA and protein expression levels of proinflammatory cytokines in the GO mouse model after treatment with the ibrutinib. Firstly, in the GO mouse model, RT-PCR revealed that the mRNA expression of proinflammatory cytokines IL-1β, IL-6, TGF-β1, IFN-γ, CD40, and CD4 was reduced by ibrutinib treatment (**Figure 7**). RT-PCR revealed lower expression levels of IL-1β (p=0.197), IL-6 (p=0.538), TGF-β1 (p=0.256), IFNγ (p=0.356), CD40 (p=0.119), and CD4 (p=0.395) in the ibrutinib-treated GO mouse model (1.04 ± 0.14, 0.44 ± 0.56, 0.19 ± 0.03, 0.27 ± 0.03, 4.24 ± 1.16, and 0.25 ± 0.03, respectively) (n=4) compared to the PBS-treated GO mouse model (1.65 ± 0.40, 0.59 ± 0.13, 0.28 ± 0.07, 0.39 ± 0.07, 8.10 ± 1.47, and 0.42 ± 0.06, respectively) (n=4) (**Figure 7**). However, no statistically significant differences were observed between the groups. Each group in this experiment consisted of 4 mice.

**Figure 8:**
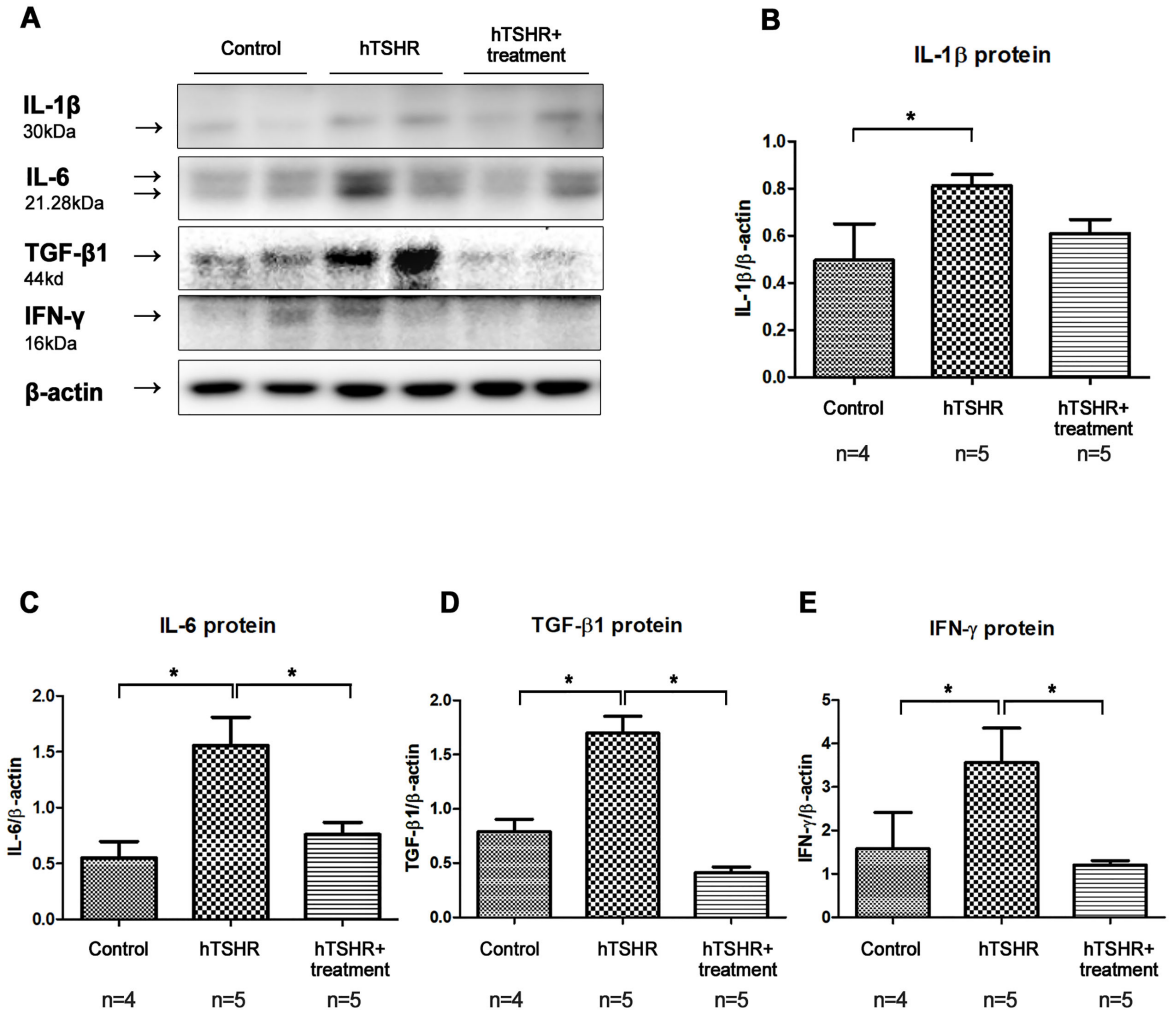
Effects of the Bruton’s tyrosine kinase (BTK) and interleukin-2-inducible T-cell kinase (ITK) inhibitor on inflammation in the Graves’ orbitopathy (GO) mouse model. **(A)** Western blotting (WB) analysis revealed elevated expression of interleukin (IL)-1β, IL-6, transforming growth factor-β1 (TGF-β1), and interferon-γ (IFN-γ) in the GO mouse model. **(B–E)** Treatment with the BTK/ITK inhibitor ibrutinib suppressed the increased expression of IL-1β, IL-6, TGF-β1, and IFN-γ in the GO mouse model. WB show 2 mice/group and are representative of 2 independent experiments with samples from 4 control mice and 5 GO mouse and 5 ibrutinib-treated GO mouse group. **(B)** Bar graphs use data from all mice per group. ^*^ *p*<0.05.

Secondly, western blotting analysis showed that the expression levels of IL-1β, IL-6, TGF-β1, and IFN-γ were higher in the GO mouse model (PBS-treated GO mice, n=5) compared to the control group (non-GO mice, n=4) (**Figure 8**). This increase was significantly suppressed by ibrutinib treatment in the GO mouse model (**Figure 8**, **Supplementary Figure 2**). The expression levels of IL-1β, IL-6, TGF-β1, and IFN-γ were lower in ibrutinib-treated GO mouse model (n=5) compared to PBS-treated GO mice (n=5) (**Figure 8**). Representative photos of two independent samples from each group (non-GO mice, PBS-treated GO mice, ibrutinib-treated GO mice) are shown (**Figure 8A**). Taken together, our results demonstrate that ibrutinib inhibits orbital inflammation in a mouse model of GO.

## 4 Discussion

BTK is a cytoplasmic kinase expressed in all hematopoietic cells except T lymphocytes and mature differentiated plasma cells, with a notable abundance in B lymphocytes. It plays a crucial role in various B-cell antigen receptor signaling pathways, influencing the differentiation of antibody-producing plasma cells and the activation, proliferation, and survival of B cells. Additionally, it is essential for the secretion of proinflammatory cytokines (25, 26). Originally identified through a mutation associated with X-linked agammaglobulinemia in human immunodeficiency, BTK is linked to inflammatory signaling (27). In autoimmune conditions such as RA and SLE, B cells are involved in the activation of innate immunity, tissue damage, and disease progression through various mechanisms, including autoantibody production, complement binding, and antigen presentation to T cells, resulting in inflammatory cytokine production and activation of cytotoxic T cells. Moreover, immune complexes formed by autoantibodies originating from B cells may deposit in target organs, triggering the activation of innate immune cells and causing tissue damage (28). Given these crucial functions, BTK is recognized as a key target for therapeutic interventions in autoimmune disorders. Recently, our study group investigated the potential role of BTK in the inflammatory pathogenesis of an *in vitro* model of GO (22). The results revealed that the BTK expression in GO orbital tissues was higher than in normal orbital tissues. Moreover, ibrutinib, an orally bioavailable BTK inhibitor, suppressed the IL-1β- and IGF-1-induced proinflammatory cytokines, such as IL-6, IL-8, and COX-2, in both GO and normal orbital fibroblasts (22).

Due to the homology between BTK and ITK, ITK, an off-target kinase, is inhibited by ibrutinib (20). ITK is a crucial cytoplasmic protein in the T-cell signaling pathway. In the present study, we compared the serum expression levels of BTK, ITK, PLC-γ1, and PLC-γ2 between GO (*n* = 15) and non-GO (*n* = 15) groups using RT-PCR. *In vivo* experiments were performed by extracting serum mRNA levels from GO patients and healthy controls. RT-PCR revealed that the baseline serum BTK, ITK, PLC-γ1, and PLC-γ2 expression levels were significantly higher in GO compared to controls. In a previous study, we demonstrated that BTK was overexpressed in orbital tissue but not in serum. Here we confirmed the involvement of BTK and ITK pathways in GO pathogenesis based on circulating serum protein levels of BTK, ITK, PLC-γ1, and PLC-γ2.

Ibrutinib is a first-generation oral BTK inhibitor that has been clinically approved by the United States Food and Drug Administration (FDA) for the treatment of various B-cell malignancies, including chronic lymphocytic leukemia and small lymphocytic lymphoma (29). Although second- and third-generation oral BTK inhibitors, such as acalabrutinib (30) and zanubrutinib (31), do not inhibit ITK and might have less ocular toxicity (32), we selected ibrutinib for this study due to the relatively extensive information available on its use in autoimmune inflammatory diseases such as RA and SLE. Moreoever, ITK also appears to be associated with GO (23). The use of ibrutinib in autoimmune and inflammatory diseases is limited by concerns about off-target safety; however, ongoing clinical trials are exploring its potential utility in these conditions (33–36). Ibrutinib has demonstrated efficacy in preventing disease development in an RA mouse model (37). In *in vitro* experiments using peripheral blood from patients with systemic sclerosis, ibrutinib reduced the production of pro-fibrotic cytokines IL-6 and tumor necrosis factor-α, while preserving the inhibitory role of IL-10, which helps suppress the overactivation of fibrotic functions in B cells (35). For the clinical application of ibrutinib in GO, ocular toxicity is a concern that should be addressed. The inflammatory complications of tyrosine kinase inhibitors are relatively well described. While ibrutinib and other kinase inhibitors are generally well tolerated, there are increasing reports of ocular toxicities, including uveitis (32). Recently, zanubrutinib has been reported to be associated with cystoid macular edema (38). Currently, corticosteroids are the mainstay treatment for GO. The European Group on Graves’ Orbitopathy guidelines recommend rituximab, a CD20 monoclonal antibody, for moderate-to-severe GO refractory to intravenous methylprednisolone pulse (39). Tocilizumab, targeting the IL-6 receptor, and sirolimus (rapamycin), an anti-proliferative and anti-fibrotic immunosuppressive drug, are considered alternative therapies (40–42). Addition of oral atorvastatin to an intravenous methylprednisolone regimen improved GO outcome in patients with moderate-to-severe, active eye disease who were hypercholesterolaemic (43). Among newer therapies, teprotumumab, an FDA-approved monoclonal antibody against the insulin-like growth factor 1 receptor, has emerged as a promising treatment (44). Teprotumumab, approved by the FDA in early 2020 for GO treatment, has demonstrated effectiveness in reducing proptosis and the clinical activity score (45–47).

In the present study, we successfully induced a GO mouse model and verified the potential therapeutic role of ibrutinib (21, 48), in GO through the inhibition of inflammatory responses. To ensure the safety of new therapeutic drugs, *in vivo* testing should be performed using animal models. Over the past 30 years, various strategies have been devised to induce key features of GD in animals. Animal models of GO offer a valuable strategy for evaluating the pathogenesis of and therapeutic approaches to the disease. Continuous efforts have been made to establish mouse models of GO. However, animal models have several limitations in terms of reproducibility and simultaneous induction of GD and GO. TSHR sequencing and the development of recombinant TSHR DNA have facilitated the establishment of animal models for GD. Moreover, recent advancements in technologies have enabled *in vivo* expression of the receptor, which is crucial for establishing the GO model (49). Recently, Berchner-Pfannschmidt et al. (50) compared the establishment of the GO mouse model in two distinct locations with comparable housing conditions. Although the cumulative incidence of orbital pathology was increased compared to control animals in both locations, hyperthyroidism was observed in only one center for unclear reasons.

Immunization against the TSHR induces *in vivo* TSHR expression through three primary approaches. In the early stages, direct administration of TSHR-expressing cells is employed; however, due to its relatively low efficiency in inducing autoimmunity, this approach is not currently utilized. Recently, genetic immunization methods have become predominant, primarily utilizing TSHR-expressing adenovirus and TSHR-encoding plasmids (51). Moreover, the success rate of genetic immunization with plasmids has been enhanced through the implementation of a specialized electroporation technique (10, 52), which leads to a sustained antibody response to TSHR, leading to a high incidence of hyperthyroidism (53, 54). Considering that the transfection efficiency may be influenced by the *in vivo* gene delivery methods, our study optimized genetic immunization through CTX administration and deeper electrode needle insertion. In the present study, all mice were administered 10 μM CTX in the biceps femoris muscle, followed by plasmid DNA administration 5 days after CTX inoculation. CTX induces significant muscle contraction and lysis of myofibers, enhancing the plasmid DNA uptake by muscle cells (55, 56). Additionally, we increased the depth of electrode needle insertion to 4 to 5 mm, ensuring that the plasmid DNA was administered at the midpoint of the line connecting the two electrode needles, with the aim of enhancing the transfection efficiency (8). In the present *in vivo* study utilizing a GO mouse model, similar to the increased BTK expression observed in our prior *in vitro* experiments in GO tissues (22), significant BTK upregulation was observed in the non-GO mouse model. Moreover, key proinflammatory mediators implicated in B cell immunity, including IL-1β, IL-6, TGF-β1, IFN-γ, CD40, and CD4, exhibited increased expression levels. These findings confirm the crucial role of BTK in the inflammatory response within GO, underscoring its significance as part of the autoimmune pathology.

In the present study, a limitation was the lack of MRI data to validate our animal model. Orbital MRI is crucial for objective quantification of adipogenesis and enlargement of extraocular muscles (EOM). Although we reported significant proptosis in half of the hTSHR plasmid-immunized mice, it is possible that more mice could be affected. This is supported by a previous clinical study indicating that a high frequency of Graves’ disease patients without clinical eye signs or symptoms exhibit EOM abnormalities detected by MRI (57). Furthermore, genetic knockdown approaches targeting BTK or ITK kinase activity using small interfering RNA would provide more specific insights for validating BTK and/or ITK as targets for future drug development, beyond the inhibition of BTK by ibrutinib. These genetic knockdown methods would enhance our understanding of the pharmacologic targets responsible for ibrutinib’s effects (58). For instance, the gene-edited C481S mouse can serve as a valuable tool to identify novel therapeutic targets and explore off-target effects associated with irreversible BTK inhibitors *in vivo* (24).

In conclusion, the GO mouse model exhibited increased BTK and ITK expression. Ibrutinib, a BTK/ITK inhibitor, suppressed inflammatory cytokine production. These findings underscore the potential involvement of BTK and ITK in the inflammatory pathogenesis of GO, suggesting that it may be a novel therapeutic target for this condition.

## Data Availability

The original contributions presented in the study are included in the article/**Supplementary Material**. Further inquiries can be directed to the corresponding author.

## Supplementary material

Supplementary Figure 1 Detail of western blots. Full-length lanes of western blot of Bruton’s tyrosine kinase (BTK), interleukin-2-inducible T-cell kinase (ITK), phospholipase C-γ (PLC-γ)1, and PLC-γ2 in the Graves’ orbitopathy (GO) mouse model. Full band revealed an increased expression level of BTK, ITK, PLC-γ1, and PLC-γ2 in the GO mouse model. Particularly, the elevated expression of BTK, ITK, PLC-γ1, and PLC-γ2 in the GO mouse model was inhibited by ibrutinib.

Supplementary Figure 2 Detail of western blots. Full-length lanes of western blot of interleukin (IL)-1β, IL-6, transforming growth factor-β1 (TGF-β1), and interferon-γ (IFN-γ) in the Graves’ orbitopathy (GO) mouse model. Full band revealed an increased expression level of IL-1β, IL-6, TGF-β1, and IFN-γ in the GO mouse model. The elevated expression of IL-6, TGF-β1, and IFN-γ, excluding IL-1β, in the GO mouse model was suppressed by ibrutinib.
