# Supplementary figures and images for: Impact of ibrutinib on inflammation in a mouse model of Graves’ orbitopathy
Source: Front Endocrinol (Lausanne). 2024 Aug 30;15:1420024. doi: 10.3389/fendo.2024.1420024 (PMC11392736; doi:10.3389/fendo.2024.1420024)

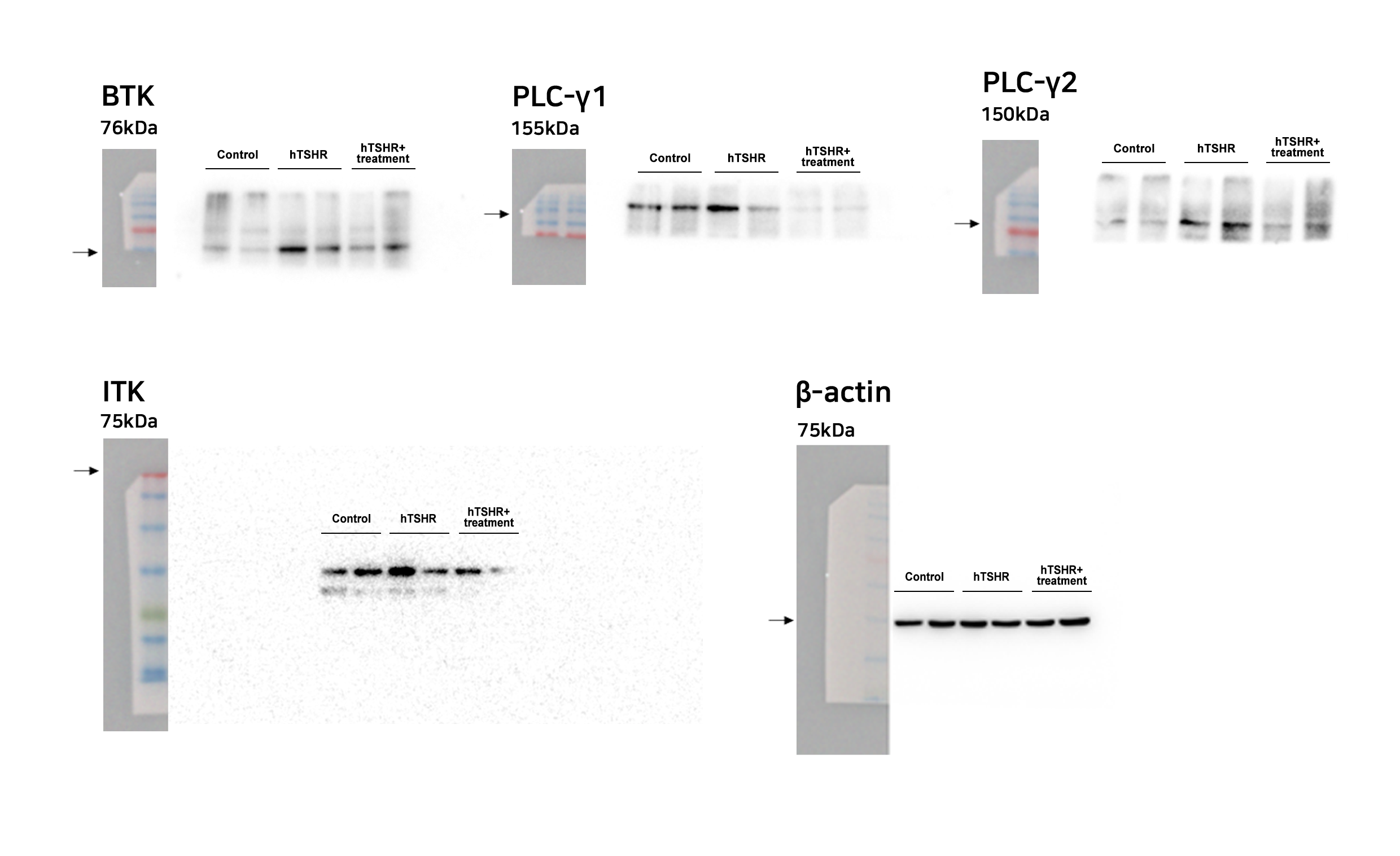

Supplement: Supplementary Figure 1 — Detail of western blots. Full-length lanes of western blot of Bruton’s tyrosine kinase (BTK), interleukin-2-inducible T-cell kinase (ITK), phospholipase C-γ (PLC-γ)1, and PLC-γ2 in the Graves’ orbitopathy (GO) mouse model. Full band revealed an increased expression level of BTK, ITK, PLC-γ1, and PLC-γ2 in the GO mouse model. Particularly, the elevated expression of BTK, ITK, PLC-γ1, and PLC-γ2 in the GO mouse model was inhibited by ibrutinib. [file Image1.tif]

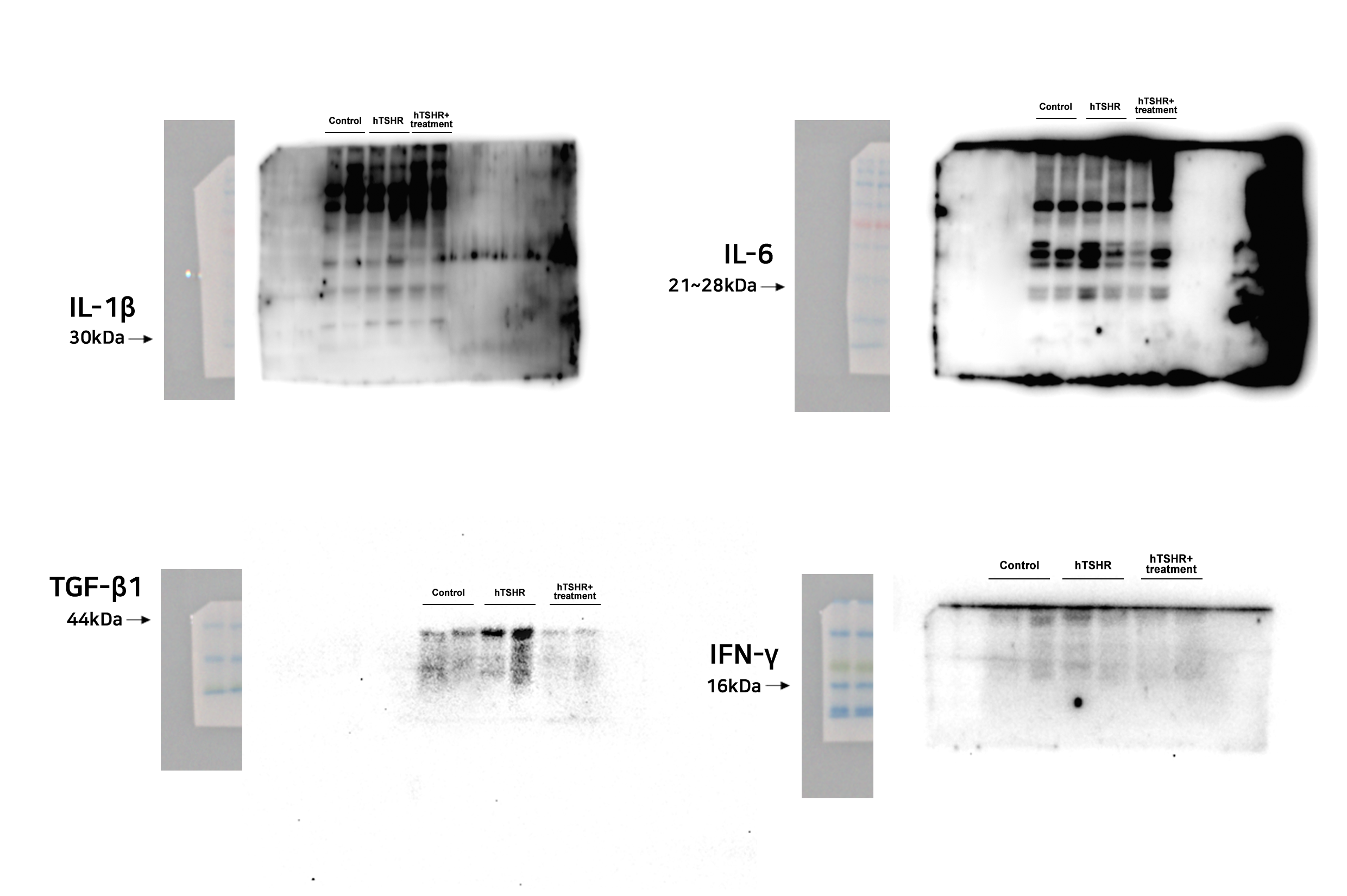

Supplement: Supplementary Figure 2 — Detail of western blots. Full-length lanes of western blot of interleukin (IL)-1β, IL-6, transforming growth factor-β1 (TGF-β1), and interferon-γ (IFN-γ) in the Graves’ orbitopathy (GO) mouse model. Full band revealed an increased expression level of IL-1β, IL-6, TGF-β1, and IFN-γ in the GO mouse model. The elevated expression of IL-6, TGF-β1, and IFN-γ, excluding IL-1β, in the GO mouse model was suppressed by ibrutinib. [file Image2.tif]
